# Alpha-chloralose poisoning in cats in three Nordic countries - the importance of secondary poisoning

**DOI:** 10.1186/s12917-022-03370-w

**Published:** 2022-09-05

**Authors:** Ulrika Windahl, Annica Tevell Åberg, Fedor Kryuchkov, Sandra Lundgren, Cecilia Tegner, Kristoffer Dreimanis, Sanna Koivisto, Outi Simola, Morten Sandvik, Aksel Bernhoft

**Affiliations:** 1 Swedish National Veterinary Institute (SVA), 75189 Uppsala, Sweden; 2 Department of Medicinal Chemistry, Analytical Pharmaceutical Chemistry, Uppsala University, P.O. Box 574, 75123 Uppsala, Sweden; 3 Toxinology Research Group, Norwegian Veterinary Institute, P.O. Box 64, NO-1431 Ås, Norway; 4 University Animal Hospital, Swedish University of Agricultural Sciences, Uppsala, 75007 Sweden; 5 Finnish Safety and Chemicals Agency, P.O. Box 66, 00521 Helsinki, Finland; 6 Finnish Food Authority, P.O. Box 200, 00027 Helsinki, Finland

**Keywords:** Alpha-chloralose, Chloralose, Poisoning, Toxicosis, Cat, Feline, Secondary, Mice, Metabolism

## Abstract

**Background:**

Alpha-chloralose (AC) is a compound known to be toxic to various animal species and humans. In 2018 and 2019 an increase in suspected cases of AC poisoning in cats related to the use of AC as a rodenticide was reported to national veterinary and chemical authorities in Finland, Norway and Sweden by veterinarians working in clinical practices in respective country. The aims of this study were to prospectively investigate AC poisoning in cats, including possible secondary poisoning by consuming poisoned mice, and to study metabolism and excretion of AC in cats through analysis of feline urine.

**Methods:**

Data on signalment, history and clinical findings were prospectively collected in Finland, Norway and Sweden from July 2020 until March of 2021 using a questionnaire which the attending veterinarian completed and submitted together with a serum sample collected from suspected feline cases of AC-poisoning. The diagnosis was confirmed by quantification of AC in serum samples. Content of AC was studied in four feline urine samples, including screening for AC metabolites by UHPLC-HRMS/MS. Bait intake and amount of AC consumed by mice was observed in wild mice during an extermination of a rodent infestation.

**Results:**

In total, 59 of 70 collected questionnaires and accompanying serum samples were included, with 127 to 70 100 ng/mL AC detected in the serum. Several tentative AC-metabolites were detected in the analysed feline urine samples, including dechlorinated and oxidated AC, several sulfate conjugates, and one glucuronic acid conjugate of AC. The calculated amount of AC ingested by each mouse was 33 to 106 mg with a mean of 61 mg.

**Conclusions:**

Clinical recognition of symptoms of AC poisoning in otherwise healthy cats roaming free outdoors and known to be rodent hunters strongly correlated with confirmation of the diagnosis through toxicological analyses of serum samples. The collected feline exposure data regarding AC show together with the calculation of the intake of bait and subsequent AC concentrations in mice that secondary poisoning from ingestion of mice is possible. The results of the screening for AC metabolites in feline urine confirm that cats excrete AC both unchanged and metabolized through dechlorination, oxidation, glucuronidation and sulfatation pathways.

**Supplementary Information:**

The online version contains supplementary material available at 10.1186/s12917-022-03370-w.

## Background

Alpha-chloralose (AC) is the alpha isomer formed by the condensation of glucose with trichloroacetaldehyde (chloral), first described in 1893 [1, 2]. It is a toxic compound currently used as a rodenticide and an anaesthetic for laboratory animals [3–5]. The exact mechanism of action of AC are unclear, but in experimental studies evaluating the toxic effect of AC, as well as case reports and from its current use as an anaesthetic for laboratory animals, the compound has been shown to have both a dose-dependent depressant and stimulant effect on the central nervous system (CNS) in various animal species and in humans [1, 4–10]. The compound is currently not used in either veterinary or human clinical practise, but older publications discussing the potential use of AC as an anaesthetic for human, feline and canine patients are available [1, 7, 11, 12].

In 2018 and 2019, a marked increase in suspected cases of AC-poisoning in cats was reported to national veterinary and chemical authorities in Finland, Norway and Sweden by veterinarians working in clinical practices in respective country [13–16]. The veterinarians suspected secondary AC-poisoning on the basis of multiple otherwise healthy outdoor cats being brought to respective veterinarian due to sudden development of neurological symptoms compatible both with the findings in publishes reports on AC-poisoning in various animal species and with the simultaneous informal reports on social media platforms from other veterinarians in clinical practice. Both the Finnish Safety and Chemicals Agency and the Norwegian Environmental Agency (NEA) reported a simultaneous increase in the sales of AC-products in the year 2018 (personal communication Sanna Koivisto, [16]. The substance was available for individual use without restrictions in Norway and Sweden until restricted for approval to professional use only in Norway by NEA from May 2020 and in Sweden by the Swedish Chemicals Agency from December 17th, 2019. In Finland a planned restriction to professional use by the Finnish Safety and Chemicals Agency in December 2021 has yet to be adopted.

Case reports summarising the clinical symptoms of AC poisoning in several animal species other than cats, including human cases with lethal outcome, are available [5–10, 17, 18]. Intoxication may lead to lethargy, somnolence, coma and/or stupor, but also hyperesthesia, muscle tremors and myoclonic seizures, sometimes with accompanying hyperthermia of unknown cause, or described as a result of increased body movement. Dose-dependent ataxia, increased salivation, and symptoms of cranial nerve disorders such as miosis or mydriasis and impaired pupillary response to light is frequently described in reports on intoxication in humans and animals. In addition, behavioural changes such as aggressiveness is reported, mainly in animals. In individuals where lethargy is present or consciousness is lost, bradycardia, hypotension, bradypnea and hypothermia may be present and if not treated or reversed in time contribute to a lethal outcome [1, 4–10].

Reports on clinical cases of AC-poisoning in cats are however rare. In 2006, Segev et al. [8] described clinical and clinicopathological characteristics in 13 cats attending the Hebrew University Veterinary Teaching Hospital with symptoms as well as anamnesis leading to suspicion of AC poisoning. In 2016 Grau-Roma et al. [4] described one case. In 2021 Dijkman et al. described in total 39 feline cases from The Netherlands, in two case series spanning the years 2014–2020 [19, 20]. In Norway, the Norwegian Veterinary Institute (NVI) verified AC-poisoning in 30 cats and four dogs in samples collected from various veterinary clinics from December 2019 to June 2020 [16], and in the year 2021, AC poisoning was confirmed in 20 individual cats using a quantitative Ultra-high Performance Liquid Chromatography tandem Mass Spectrometry (UHPLC-MS-MS) method for analyses of serum samples [21]. Peer-reviewed scientific reports with quantitative laboratory analyses of the substance in blood, urine, or other tissues from cases of AC-poisoning in animals as well as in humans are otherwise rare. To the best of our knowledge, presently the study published in 2021 by Windahl et al. [21] is so far the only previous study using a validated quantitative analytical method for detection of AC in feline blood samples published in peer-reviewed literature.

The toxicity of AC including minimal lethal dose is expected to vary between different species. Minimal lethal dose in dogs has been reported to be approximately 600–1000 mg/kg, for rats and mice 400 and 300 mg/kg, respectively, while the minimal lethal dose in cats has been reported to be considerably lower: 100 mg/kg [1, 8, 11, 12, 22]. For birds such as starlings and crows even lower mean lethal oral doses (LD_50_) are reported: 76 mg/kg and 42 mg/kg respectively [1, 8, 11, 22]. The therapeutic index in cats has to the best of our knowledge never been fully determined, although reports on use of AC as a feline anaesthetic are available. For example, Kullman et al. used continuous intravenous infusion of AC at a dose of 5 mg/kg/h after an initial bolus of 65-75 mg/kg as anaesthesia in cats used for experimental research not related to AC toxicosis [3].

The exact metabolic pathways of AC are unclear [5, 8, 23, 24]. After ingestion or injection AC is hydrolysed to chloral [1, 8]. Further reduction to the CNS depressant metabolite trichlorethanol has been described [1, 8], as has conjugation of chloral with glucuronic acid in the liver resulting in the inactive urochloralic acid being excreted by the kidneys [8, 25]. However, some researchers report excretion of chloralose, either mainly unchanged or metabolized as a glucuronide [6, 8, 26]. This included the two case studies by Thomas et al. [6] where trichlorethanol was not detected in neither blood nor urine samples from humans exposed to AC, although AC was detected in the same samples. These findings led the authors to doubt the role of trichlorethanol in the metabolism and action of AC, at least in human patients. The possibility of trichlorethanol instead being oxidized to trichloroacetic acid has also been discussed [8, 27].

Cats are known to have a limited capacity to form glucuronide metabolites for drugs compared to other species [28, 29]. Glucuronic acid conjugation is a metabolic process that increases the polarity of xenobiotics and hence facilitates the excretion of several compounds including drugs and toxins via urine or bile. While the human UDP-glucuronosyltransferase gene (UGT1A) codes for nine different UGT enzymes, and the canine gene ten, the feline gene only encodes two different UGT enzymes that catalyses the conjugation with glucuronic acid, thereby leading to reduced excretion of substances secreted by this mechanism [29]. Cats are also deficient in other enzymes responsible for conjugation reaction, e.g., N-acetyltransferase and thiopurine methyltransferase [29]. Bernhoft et al. [16] reported results that indicated marginal UGT conjugation of AC in feline urine with their method that analysed both free and total AC after deconjugation with ß-glucorindase. However, peer-reviewed studies on metabolites of AC in feline urine is to the best of our knowledge lacking.

 In conclusion, peer-reviewed publications describing AC-poisoning in cats and studies investigating possible secondary AC-poisoning in wild and domestic animals are currently rare. The present study was initiated due to a marked increase in reports of suspected cases of AC-poisoning of cats in Finland, Norway and Sweden, and an accompanying awareness of the potential risks of an unrecognised increase of secondary poisoning in cats as well as also in wild carnivores and birds, including birds of prey. The aims were to investigate possible AC-poisoning in cats through analysis of presence of AC in blood samples collected from outdoor cats with relevant clinical symptoms using a validated quantitative analytical method, combined with the collection of relevant anamnestic data using a standardised questionnaire. The possibility of secondary AC-poisoning via intake of mice was to be studied further by recording the amount of ingested commercial AC-rodenticide by wild mice (*Apodemus flavicollis;* Yellow-necked field mouse). Finally, the metabolism and urine excretion of AC in cats was to be studied through qualitative and quantitative analysis of feline urine.

## Methods

### Feline cases

Data on signalment, history and clinical findings in suspected feline cases of AC-poisoning and accompanying serum samples for toxicological analyses were prospectively collected in Finland, Norway and Sweden from July 2020 until March of 2021 using a questionnaire (Table 1). Inclusion criteria were sudden onset of clinical symptoms consistent with previously published cases of AC poisoning, including two or more relevant neurological symptoms in previously healthy cats allowed to roam free outdoors. Cases fitting the inclusion criteria on the basis on collected data were to be excluded if AC was not detected by the subsequent chemical analyses of the accompanying serum sample.

**Table 1:** Anamnestic questions, the pre-determined response categories and open questions included in the Questionnaire

Questions	Pre-determined response categories
Is the cat regularly roaming free outdoors?	Yes; No
If Yes:	Suburb; City; Countryside
Had the cat roamed free outdoors within approximately 24 h prior to onset of symptoms?	Yes; No; Probably; Unknown
Location of the cat at time of discovery of symptoms	Indoors; Outdoors; Unknown; Other
Is the cat a known rodent hunter?	Yes; No
Did the owner see the cat ingesting rodent/rodents on the day of suspected poisoning?	Yes; No
Were there dead rodents, or visibly somnolent rodents near where the cat roams on the day of the acute debut of symptoms?	Yes; No; Unsure; Do not know
If yes: Where were the dead rodents, or visibly somnolent rodents?	Inside a bait-box; Outside of a bait-box; Possible presence of rodent trap unknown
Is rodenticide used near the home of the cat?	Yes; No; Unsure; Do not know
If yes:	Indoors in home/stable where the cat lives; Outdoors; Neighbours; Other
If yes: which poison(s)/ product(s):	(Free text)
Did the owner see the cat ingesting rodenticide?	Yes; No; Unsure
Any additional information as to how the cat came into contact with alpha chloralose?	(Free text)
On what grounds were alpha-chloralose poisoning suspected?	Anamnesis combined with symptoms; Symptoms, Knowledge of the feline cases of alpha chloralose poisoning in the area; Other
Were there other, differential diagnosis initially?	Yes; No; They are still relevant, diagnosis not clear
If yes, please name the differential diagnosis	(Free text)
Was abdominal diagnostic imaging performed?	Yes; No
If yes: Was mice or remnants of mice detected?	Yes; No

The Finnish Food Authority used veterinary social media platforms to recruit veterinary clinicians, while the Norwegian Veterinary Institute (NVI) recruited veterinarians via the institute’s website. Veterinarians were requested to complete and submit the questionnaire together with an owner consent and leftover serum from routine blood samples collected from feline patients with suspected AC poisoning. In Norway, veterinarians were also asked to submit a leftover urine sample if such a sample was available. The veterinary clinicians were provided with written instructions for sample handling and shipment as well as an owner consent document and the questionnaire from the respective authority. The samples were stored in − 20 ^o^C by the Finnish Food Authority, Unit of Bacteriology and the Pathology and the Toxinology Research Group at NVI respectively, prior to further shipment for analysis to the Swedish National Veterinary Institute (SVA). In Sweden, veterinary clinicians employed at the Swedish University Animal Hospital (UDS) stored left over sera in − 20^o^C in a biobank at UDS prior to shipment for analysis to SVA and transferred clinical data from relevant feline patients’ charts to the designated questionnaire.

The questionnaire mainly consisted of closed-ended, multiple choice questions, but for some questions the responders were also encouraged to add free text if further information was available. The latter included the request for any additional information as to how the cat came into contact with AC. Open questions with free text answers were specifically used for brand names of rodenticides, medication used and for listing potential differential diagnosis. In short, in the first section of the questionnaire, the veterinarian was asked to respond to predetermined questions on the cats signalment; name, age, breed, weight and sex (male castrated, female castrated, female entire or male entire), as well as the home address of the cat and the name of the responding veterinarian and the veterinary clinic. The second section concerned anamnestic questions including hunting behaviour, possible or known access and ingestion of rodents as well as possible and known or suspected presence of, and exposure to, AC-based rodenticides (Table 1). The third section concerned clinical findings. Relevant symptoms were listed in the questionnaire, and the veterinarians were asked to mark all symptoms applicable to respective cat (Table 2). In addition, questions on recovery or death were included. Furthermore, the veterinarians were asked to describe additional investigations performed at the clinic and what kind of medical treatment the cat received. The data collected in the questionnaires was compiled and assessed at SVA using Microsoft Excel (Microsoft Corporation, 2018. Microsoft *Excel*, available at https://office.microsoft.com/excel. All data was collected and stored according to national law and the General Data Protection Regulation (GDPR) EU 2016/679.

**Table 2:** An overview of clinical findings listed in the questionnaire and the number and percentages of responders reporting each symptom

Clinical findings^a^	Percentage of cases^b^
Tremor	93% (*n* = 55)
Ataxia	72% (*n* = 43)
Hyperesthesia	69% (*n* = 41)
Seizures	12% (*n* = 7)
Somnolence	42% (*n* = 25)
Stupor	10% (*n* = 6)
Coma	3% (*n* = 2)
Bradycardia^c^	39% (*n* = 23)
Hypothermia^d^	35% (*n* = 21)
Miosis, Mydriasis, Anisocoria, Impaired Pupillary light reflex, Impaired menace response, Impaired dazzle- response^e^	58% (*n* = 34)
Visual impairment or blindness	44% (*n* = 26)
Behavioural changes	39% (*n* = 23)
Ptyalism	5% (*n* = 3)

^a^ Clinical finding listed in the questionnaire

^b^ Percentages of the 59 included cases where the finding was reported

^c^ Defined as a heart rate of ≤ 140/min

^d^ Defined as a body temperature of ≤ 37 °C

^e^ One or several of the listed findings noted in respective report

A validated quantitative analysis was used to analyse the collected serum samples, specifically the UHPLC – MS/MS method described by Windahl et al. [17]. In short, the serum proteins were precipitated by acetonitrile and the samples were diluted with water before UHPLC–MS/MS analysis. The AC detection limit of the method was 30 ng/mL, and the limit of quantification was 100 ng/mL.

### Quantitative analyses of alpha-chloralose content in ingested mice

Two of the cats included in the study (cat number 14 and 17) vomited during the veterinary visit, with the contents including one mouse each, which were sent to the NVI for analyses. After preparation of the mice-samples as described in the supplementary material in SI3, samples were analysed using the UHPLC-MS/MS instrument (supplementary material, SI). The amount of AC in samples A and B were calculated by constructing corresponding calibration curves using the standard addition to extracts approach (Figs. S1-S2).

### Bait intake in mice

Alpha-chloralose intake by 14 wild mice; *Apodemus flavicollis* (Yellow-necked field mouse), and the commercial bait containing AC at a concentration of 40 mg/g (Trinol dry No mouse korn® alpha-choralose Casnr.15879-93-3, LODIS A.S F-35,390, Grand Fougeray, France) was observed in mice caught in traps set up indoors in a cabin during an extermination of an infestation of wild rodents in a cabin in Viken, Norway during October 2020. The mice were caught in one trap each. The duration of feeding time and time from start of feeding to death was recorded for each mouse. The amount of bait remaining in the trap, as well as the individual mouse, was weighed after the death of each mouse. The amount of bait relative to the body weight of the mouse was calculated as well as the amount (mg) of AC ingested.

### Urine analyses

#### Preparation of urine samples for semiquantitative analysis of alpha-chloralose

Urine collected by the attending veterinary clinician was available from in total ten cats (cat number 28–30, 32–34, 39, 40, 43, and 48) for investigation of urine excretion of AC. Out of these ten, four were selected for investigation of the metabolism of AC prior to urine excretion (cat number 28, 32, 34, and 43). All ten urine samples were analysed prior to the assessment of collected questionnaire data and chemical analyses of the serum samples.

The urine samples were stored at the NVI in − 20 °C. Prior to analyses the samples were thawed and a 250 µL aliquot of each sample were mixed in polypropylene tubes with 750 µL of chloroform. Organic phase was dried off in nitrogen flow and suspended in 3:1 mixture of acetonitrile and 10 mM solution of ammonium acetate in water (v/v). Insoluble inorganic content was separated by centrifugation (20,000 rcf for 5 min) and clear supernatants were transferred to HPLC vials. All solvents and chemicals used were of analytical grade or better and purchased from Sigma Aldrich (St. Louis, MO, USA). The samples were analysed on a UHPLC–MS/MS instrument (detailed conditions are described in SI1).

#### Preparation of urine samples for screening for alpha–chloralose metabolites

The four urine samples that contained a high level of AC (cat number 28, 32, 34, and 43) were further analysed to screen for chloralose metabolites. A 250 µL aliquot of each thawed sample were mixed with 750 µL of methanol in polypropylene tubes, vortex for 1 min and left in – 20 °C for 1 h. Precipitated proteins and insoluble inorganic content were separated by centrifugation (19,600 rcf for 5 min) and clear supernatants were transferred to HPLC vials. These samples were analysed by UHPLC– HRMS/MS (detailed conditions are described in SI2). Raw data from the UHPLC–HRMS/MS analysis of the feline urine samples were analysed for the presence of phase I and phase II metabolites of chloralose by searching for structures with chemical formula from C_7_H_9_Cl_2_O_5_ to C_15_H_20_Cl_3_O_14_S within an MS tolerance of 5 ppm.

## Results

### Feline cases

**Fig. 1:**
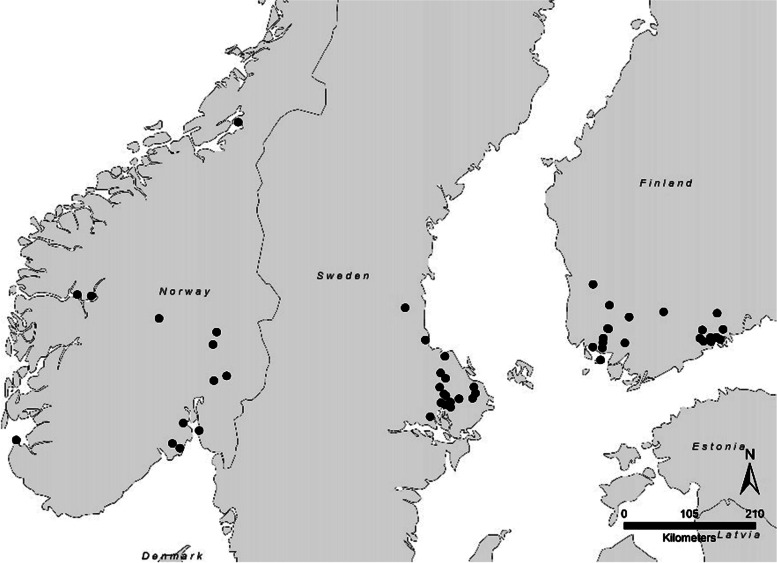
The geographical distribution of the in total 59 included cases

In total, 70 questionnaires and sera were collected during the study period. Eleven of the 70 cases were excluded due to either the submitted data in the questionnaires not being in line with the predetermined inclusion criteria (*n* = 7), or due to AC not being detected in the serum samples (*n* = 4). However, by mistake, the blood samples of the seven already excluded cases were later analysed. No AC was detected in any of the seven samples, leaving all eleven excluded cats AC– negative. Three of the excluded cases were Finnish and eight Norwegian. The geographical distribution of the 59 cases included in this study is illustrated in Fig. 1. Out of the 59 cases, 22 were from Finland, 22 from Sweden, and 15 from Norway.

The mean weight of the cats was 4.7 kg, with the majority of cats 78% (*n* = 46) weighing 5 kg or less. Specifically, 41% (*n* = 24) of the 59 included cats weighed between 4 and 5 kg, and (37%, *n* = 22) weighed less than 4 kg. Of the remaining 22% (*n* = 13), all but one weighed between 5.1 to seven kg. Breed was reported for 58 cats. In 97% (*n* = 56) of the cases, the breed was reported as either domestic shorthair (71%, *n* = 41) or as domestic mixed breed (*n* = 13). Only two cats (3%) were reported to be purebred cats (Norwegian Forest cat). Most of the cats, 85% (*n* = 50) were neutered. Eight of the nine intact cats were female. Age was reported for 58 of the 59 included cats. In total 75% (*n* = 44) were within the age span one to ten years old. 12% (n = 7) were between six– and ten months old, leaving seven cats (12%) older than ten years.

All 59 cats were reported to have been roaming free outdoors, both regularly and within approximately 24 h prior to onset of symptoms (Table 3). In total 86% (*n* = 51) were known to be rodent hunters. In an additional 10% (*n* = 6) of cases rodent consumption on the day of the symptoms was known. In four of these cases, including one cat not known to be a rodent hunter, the owner had either noted that respective cat consumed a rodent, or observed rodents outdoors on the day in question (Table 3). Two other cats not known to be rodent hunters and not spotted consuming mice vomited one dead mouse each during the veterinary visit (Table 3).

**Table 3:** An overview of reported anamnestic data for the 59 included cases

**Access to rodents** *Percentage and number of the included 59 reports*	
The cat was free roaming outdoors within 24 h of onset of symptoms.	100% (*n* = 59)
The cat was either a known rodent hunter or consumption of mice at the of development of symptoms was known.	96% (*n* = 57)
**The cat was a known rodent hunter, subcategories** *Percentages presented are of the 57 cases where the cat was a known rodent hunter*	
The cat was spotted consuming a rodent or rodents on the day of onset of symptoms,	7% (*n* = 4)
The cat regurgitated a mouse during the veterinary visit.	3.5% (*n* = 2)
**Suspicion or knowledge of use of rodenticides in the cats´ outdoor living environment** ^a^ *Percentage and number of reports where data was included*	
The cat owner was unsure of presence of rodenticide products and had no knowledge of either other cats being poisoned, or of presence of dead or somnolent mice.	46% (*n* = 27)
The cat owner reported knowledge of a rodenticide product or products being used in the cats**´** outdoor living environment^a^	32% (*n* = 19)
Reports included either knowledge of poisoning of cats, or presence of dead or somnolent mice being noted in the cats**´** outdoor living environment.	22% (*n* = 13)
**Suspicion or knowledge of use of rodenticides near the home of the cat, subcategories**	
Mice regurgitated during the veterinary visit contained AC.	*n* = 2
Knowledge of the cat consuming AC- based rodenticide mixed in cat food reported.	*n* = 1
The veterinarian reported seeing the same cat due to similar symptoms on two separate occasions, with recovery in between.	*n* = 2
Two owners had two of their cats developing similar symptoms during the course of one day.	*n* = 2
The owner reported knowledge of other cats being poisoned with similar symptoms in the neighbourhood	*n* = 1
Owner reported noting either dead or visibly somnolent rodents near the home of the cat on the day of onset of symptoms.	*n* = 5

^a^ Including use of rodenticides in buildings to which the cat had access, such as stables for large animals

Approximately a third (32% *n* = 19) of the responders reported knowledge of use of rodenticide in the cats´ outdoor living environment, including in buildings to which the cat had access such as stables for large animals. Knowledge that the rodenticide used was an alpha–chloralose based product was included in seven of these 19 reports. Approximately a fifth (22%, *n* = 13) of the cat owners who reported that they were unsure of potential use of rodenticide simultaneously reported either knowledge of other cats being poisoned, their own cats showing symptoms of poisoning more than once, or presence of dead or somnolent mice (Table 3). In the remaining 46% (*n* = 27) of the reports the cat owners were unsure of whether rodenticide products were present in the neighbourhood or not, and information on suspicion of other feline cases was lacking. The 13 reports where additional information on suspicion of AC being present in the cats´ outdoor living environment was reported included two cats belonging to the same owner developing similar symptoms during the course of one day. In two other cases, the veterinarian reported attending the same cat twice due to development of similar symptoms, with recovery in between the visits. Samples were submitted from one of the visits of respective cat. Another responder reported knowledge of unspecified cases of cats being poisoned in the neighbourhood. Five cat owners had noted either dead or visibly somnolent rodents near their home on the day when the cat fell ill, of which three reported detecting the mice outside of a baiting box. Also included in the 13 reports are the two cases mentioned above, where two cats vomited a mouse during the veterinary visit. The mice were sent to the NVI for analyses, and AC was detected. Furthermore, one responder had found the cat seizuring next to a bowl of cat food where an AC– based rodenticide had been mixed in with the food (Table 3).

The outdoor environment where the cats roamed was categorized as countryside by 80% (*n* = 47) of the cat owners, as suburb by 19% (*n* = 11), and by one as city– environment. The percentage of owners discovering the symptoms indoors versus outdoors was similar; 49% (*n* = 29) and 47% (*n* = 28), respectively. For two cats such data was missing.

Symptoms reported by the responders are shown in Table 2. Tremor, ataxia, and hyperesthesia were noted in 93% (*n* = 55), 72% (*n* = 43), and 69% (*n* = 41) of the 59 reports, respectively. In approximately half of the cases (56%, *n* = 33) somnolence, stupor or coma was reported. Bradycardia, defined as a heart rate ≤ 140/min and hypothermia, defined as a body temperature of ≤ 37 °C was reported in 39% (*n* = 23) and 35% (*n* = 21) of cases. Impaired pupillary reflexes, and/or miosis or mydriasis was reported in 58% (*n* = 34) cases, and visual impairment in 44% (*n* = 26) of the 59 included cases. Reports on behavioural changes in fully conscious cats varied notably between the three countries. Sweden, i.e., UDS reported such findings in 72% (*n* = 16) of their cases compared to a third of the Norwegian reports (33%, *n* = 5) and 9% of the Finnish reports (*n* = 2). Data on recovery or death was included by 56 responders. One cat died within two days of onset of symptoms. Of the remaining 98% (*n* = 55) recovered within one to four days after onset of symptoms. Data on plasma biochemistry investigations and on specific treatment was so sparsely reported that no overall conclusion could be drawn.

**Fig. 2:**
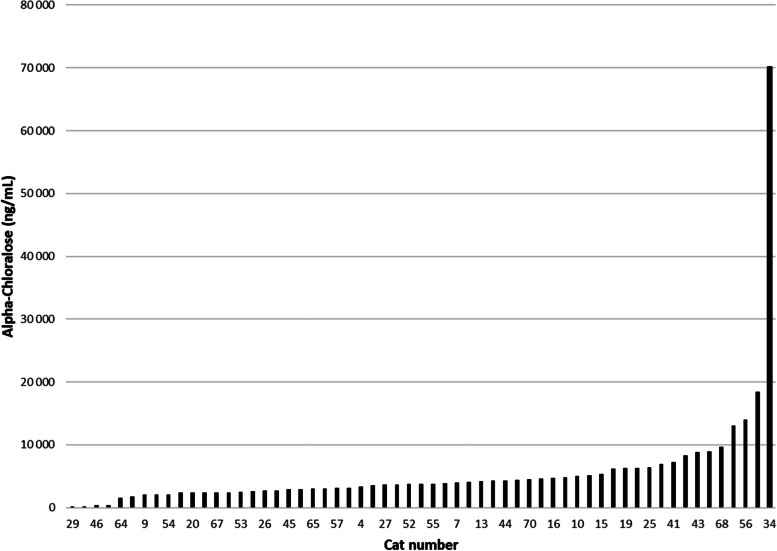
Alpha-chloralose concentration in 59 serum samples from cats with suspected AC-poisoning collected in Finland, Sweden, and Norway

The serum concentration of AC in the 59 included feline cases is shown in Fig. 2. Alpha–chloralose was quantified in all samples, the lowest concentration was 127 ng/mL and the highest 70 100 ng/mL. The mean and median concentrations were 5 597 and 3 740 ng/mL, respectively. Alpha–chloralose could not be detected in serum from any of the 11 cats that did not meet the criteria for AC– poisoning and hence were excluded from the study.

### Quantitative analyses of alpha–chloralose content in ingested mice

Alpha–chloralose was detected in both of the vomited mice. The weight of each of the two partially digested mice was 13.6 g (cat number 14) and 17.4 g (cat number 17). The total concentration of chloralose in stomach content was 279, and 17 mg/kg, respectively (Figs. S1– S2). The total mass of chloralose per mice were 3 797 and 298 µg, respectively.

### Bait intake in mice

All of the caught mice started consuming the bait within 15 min of entering the trap. The timespan from start to end of feeding was ten to fifteen minutes. All mice died within 30 min after start of feeding. The calculated amount of bait (Trinol dry No mouse korn® alpha-choralose Casnr.15879-93-3, LODIS A.S F-35,390, Grand Fougeray, France) ingested by the 14 wild mice observed during an extermination of a rodent infestation varied from 0.82 to 2.64 g, with a mean of 1.51 g. All of the 14 mice died after ingestion. Four mice consumed all the available bait. The amount of ingested bait related to body weight varied from 3.9 to 13.7%, with a mean of 8.4%, with five mice consuming more than 10% related to their body weight. The concentration of AC in the commercial baits was 4%. The calculated amount of AC ingested per mouse spanned from 32.8 mg to 105.6 mg, with a mean of 60.6 mg (Table 4).

**Table 4:** Amount of consumed rodenticide bait Trinol No mouse korn® alpha-choralose 40 mg/g in 14 wild mice (*Apodemus flavicollis*; Yellow-necked fieldmouse) caught in mousetraps, and calculated intake of AC for each mouse

Mouse ID (number)	Body weight (g) after death	Bait placed in mousetrap (g)	Bait left in trap (g)	Bait consumed by mouse (g)	Bait consumed: mouse body weight (%)	AC consumed (mg/mouse)
1	16.2	2.02	0	2.02	12.5	80.8
2	15.8	2.04	0	2.04	12.9	81.6
3	22.5	1.81	0	1.81	8.0	72.4
4	16.8	10.24	8.01	2.23	13.3	89.2
5	20.8	10.47	9.65	0.82	3.9	32.8
6	23.1	10.18	8.90	1.29	5.6	51.6
7	14.5	10.04	9.14	0.90	6.2	36.0
8	15.5	2.13	0	2.13	13.7	85.2
9	18.3	1.98	1.12	0.86	4.7	34.4
10	15.1	2.34	1.19	1.15	7.6	46.0
11	21.6	10.14	7.50	2.64	12.2	105.6
12	24.0	10.09	8.51	1.58	6.6	63.2
13	21.8	10.03	9.16	0.88	4.0	35.2
14	12.3	9.80	8.93	0.86	7.0	34.4
Mean	**17.7**	**-**	**-**	**1.51**	**8.4**	**60.6**

### Urine analyses

#### Semiquantitative analysis of alpha– chloralose

Alpha–chloralose was detected in seven of the ten available urine samples analysed for the presence of the compound. The negative urine samples were from cats where the subsequent chemical analysis of serum samples did not detect AC either (Table 5). Overall, the estimated levels of AC in the urine samples corresponded well to the AC–levels in the serum samples-.

**Table 5:** Results from the alpha-chloralose (AC) analyses of serum (quantitative) and urine (semiquantitative) from the ten cats where urine samples were available

Cat number	AC in urine (ng/mL)	AC in serum (ng/mL)
34	90 000	70 100
43	60 000	8 800
32	60 000	4 845
28	40 000	18 400
40	6000	166
39	5000	5 150
29	<LOQ = 100 ng/ml	127
30	<LOD = 30 ng/ml	<LOD = 30 ng/ml
33	<LOD = 30 ng/ml	<LOD = 30 ng/ml
48	<LOD = 30 ng/ml	<LOD = 30 ng/ml

#### Screening for alpha–chloralose metabolites

**Fig. 3:**
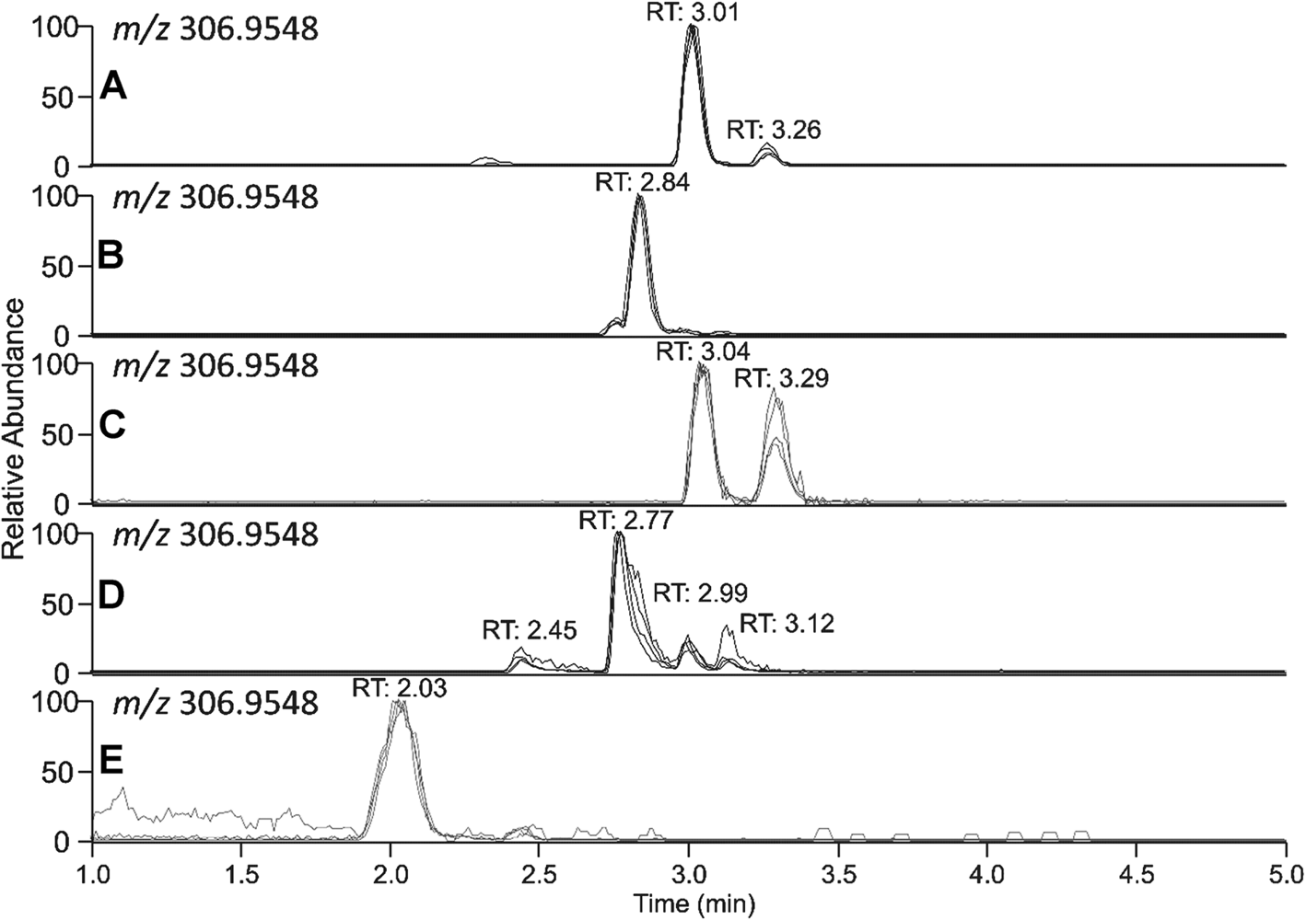
Extracted ion chromatograms from UHPLC–HRMS analysis of one of the urine samples showing the presence of several AC-metabolites

Several tentative AC–metabolites were detected in urine based on their accurate masses following UHPLC–HRMS analysis (Table 6). Data was evaluated using the Compound Discoverer software followed by manual verification. The extracted ion chromatograms were subsequently plotted (Fig. 3) for four of the feline urine samples. Significant amounts of AC were excreted unchanged in the urine, and some metabolites existed as different isomers. In total, four different isomers of sulfate phase II metabolites and one glucuronidated phase II metabolite were detected as well as one dechlorinated and two oxidated phase I metabolites. To further confirm the tentative structures of the metabolites, target m/z values were added to the inclusion list of PRM experiment and their UHPLC– HRMS/MS spectra were acquired (Figs. S3– S7).

**Table 6:** Alpha-chloralose metabolites identified by the Compound Discoverer software and their chemical formulas, m/z, delta mass, and retention time

Tentative structure	Formula	m/z	Δ mass (ppm)	Retention time (min)
Chloralose	C_8_H_11_Cl_3_O_6_	306.9548	1.00	3.01 (α), 3.26 (β)
Chloralose glucuronide	C_14_H_19_Cl_3_O_12_	482.9869	0.52	2.84
Chloralose oxidation	C_8_H_9_Cl_3_O_7_	320.9341	-0.24	3.04, 3.29
Chloralose sulphate	C_8_H_11_Cl_3_O_9_S	386.9117	1.37	2.45, 2.77, 2.99, 3.12
Chloralose de-Cl	C_8_H_12_Cl_2_O_9_	272.9938	0.80	2.03

## Discussion

The clinical suspicion of AC–intoxication based on a healthy cat roaming free outdoors suddenly developing typical neurological symptoms correlated well with presence of AC in blood samples. This supports not only the choice of selected inclusion criteria, but also the diagnosis AC–intoxication. Cats with other diseases could be expected to have different outcomes and reports of clinical symptoms differing to a higher degree between attending veterinarians in a multicentre study involving several veterinary clinics and clinicians. Furthermore, this highlights the possibility of recognition of AC intoxication as a primary differential diagnosis based on the clinical presentation and anamnesis of respective cat. The 100% inclusion rate of Swedish cats could be due to the fact that the university hospital prior to the current study had approximately eighty suspected, and 25 other confirmed feline cases admitted from January 2014 – February 2020 [21]. Such clinical experience is helpful in identifying strongly suspected cases. Notably, the percentage of symptoms recorded, as well as the survival rate, were similar between the three countries, with the exception of percentages of reports including behavioural changes. As the main difference in reports including behavioural changes was between Sweden, where this was reported in 72% of cases, and Norway and Finland (33% and 9%, respectively), one possible explanation could be that the difference was due to the Swedish cats being treated at an animal hospital where all symptoms were recorded continuously in patients charts over more than one day before being transferred to the questionnaire data. In all, the results of the study indicate that there was a good correlation between clinical suspicion and correct diagnosis in all three countries.

As the time from consumption of the toxin to respective cat being presented to the attending veterinarian is unknown, a possible correlation between reported symptoms and concentrations detected was not the focus of the study. Time to recovery was however reported in 95% of the included cases, and only one cat, with the lowest serum concentration of all cats at time of sampling (127ng/ml, cat no 29) died. These results indicates that not even the highest concentrations detected in the present study, up to 70 100 ng/mL, can by default be considered lethal, despite the current lack of available specific treatment such as an antidote. The mean and median concentrations were 5 597 and 3 740 ng/mL. In a previous study, a range of 538 to 17 500 ng/mL were recorded in 20 cats admitted to UDS due to AC-poisoning, all of which survived [21]. As the present study was not primarily designed to evaluate efficacy of treatment strategies, and as mentioned above, data on treatment was only sparsely reported, no conclusions could be drawn on the importance of different treatment strategies.

In almost all (96%) of the 59 included cases the cat was either reported to be a known rodent hunter or was known to have consumed mice prior to development of symptoms of intoxication. The latter; consumption of mice, was known in 10% through the owner observing this on the day of consumption (*n* = 4) or through the cats vomiting mice containing AC during the veterinary visit (*n* = 2). These results do not directly prove that the majority of cases included in the study are cases of secondary poisoning through ingestion of mice. However, together with the calculation of the contents of AC in mice, the results show the possibility of such secondary poisoning occurring. Notably, even if intoxication through direct ingestion of bait instead of mice is suspected, the availability of either bait or intoxicated mice outside of the traps is in itself a problem for susceptible animal and bird species. The knowledge of either poisoning of other cats in the neighbourhood, or of presence of dead or somnolent mice in the cats´ outdoor living environment reported by approximately a fifth (22%) of the responders indicate that non–domesticated animals were also at risk of AC-poisoning during the study period.

As the expected daily intake of dried feed in laboratory mice is approximately 12% of their body weight [30], the amount of AC–containing bait consumed by the wild mice after being trapped in the present study is equivalent to the expected daily amount feed intake. The concentration of AC in the commercial baits used in all three countries at the time of the study was 4% [31]. Notably, the calculated amount of AC ingested by each mouse (33–106 mg with a mean of 61 mg) corresponding to approximately 1650– 5300 mg/kg, is higher than the published lethal dose for mice of 300 mg/kg [1, 8, 11, 12, 22]. The mean intake of AC, 61 mg, corresponds to approximately ten times the expected LD_50_. The calculated amount of AC consumed per kg bodyweight in a cat weighing three kg ingesting either a mouse containing the lower calculated dose of 33 mg or the highest 106 mg, is 11 mg/kg or 35 mg/kg, respectively. These doses are both higher than the continuous infusion of AC at a dose of 5 mg/kg/h used as anaesthetic dose for cats by Kullman et al. [32]. Also, clinical symptoms are to be expected to present at lower doses than the reported lethal dose of 100 mg/kg for cats. Furthermore, cats can eat more than one mouse. Cats not fed by humans kill, and probably consume, in average about one mammal per day, consisting of mice, voles and others [33, 34]. In addition, owners of cats included in the present study described somnolent and dead mice being detected outside of traps i.e., mice more easily caught and consumed by cats and other carnivores.

The increase in reports of suspected cases of AC-poisoning to national veterinary and chemical authorities in Finland, Norway and Sweden by veterinarians working in clinical practices during 2018 and 2019 were almost all reports involving cats, despite other carnivores including dogs may also consume rodents, and/or AC–containing bait and the compound is known to be toxic for other species, including for example birds and hedgehogs as well as sheep and pigs [1, 22]. The comparatively lower reporting to the authorities of suspected cases in dogs might be due to different exposure conditions as well as the differences in drug metabolism [8, 16]. Cats might be more prone to secondary poisoning by eating poisoned birds or rodents [8] and non–hunting, smaller dogs might more often be under surveillance of the owner while outdoors. Importantly, the lethal dose in dogs has been reported to be approximately six to ten times higher than for cats [1, 11, 22]. The required dose for noticeable, lingering symptoms to develop in dogs can therefore be expected to be higher than for cats, including for small, cat–sized dogs. Unlike wild animals, many cats have close contact with owners who detect the symptoms and bring the cat to a veterinary practice. Cats might therefore in sense be regarded as sporadic sentinels for risk of AC intoxication in wild birds and smaller carnivores, regardless of whether respective cat has consumed AC–containing bait or via poisoned birds or mice.

As stated above, the exact metabolic pathways of AC are unclear [5, 8, 23, 24], and peer–reviewed studies on metabolites of AC in feline urine are lacking. In this study, several novel sulfate metabolites were detected, as well as metabolites formed by oxidation or dechlorination reactions, which were all assigned based on their HRMS and HRMS/MS spectra. To the best of our knowledge, this is the first report of presence of multiple metabolites of AC in urine samples. In addition, the detection of one glucuronic acid conjugate of AC indicates that glucuronidation of AC might be possible in cats, even though cats are known to have a limited capacity to form glucuronide metabolites of drugs [28, 29]. Some of the AC was excreted unmodified in the urine, but accurate quantification of the metabolites could not be performed without authentic standards, which were not commercially available. Furthermore, the results might be influenced by rodent metabolism, if the cats were subjected to secondary poisoning. Further investigations of the relevance of various metabolites of the compound in relation to development of clinical disease and death in various animal species including in cats are warranted.

## Conclusions

The suspicion of AC intoxication based on a healthy cat suddenly developing typical neurological symptoms corresponding to previous publications of AC poisoning was in this study confirmed by quantification of serum concentration of AC. A strong correlation was shown in all three countries between clinical recognition of symptoms and confirmation of the diagnosis.

The collected data on exposure to AC together with the calculation of the intake of bait and subsequent AC concentrations expected in mice show that secondary poisoning from ingestion of mice is possible. Results of the study also highlight the risk of AC poisoning to non-target species.

As one glucuronic acid conjugate of AC was detected in feline urine from feline cases of AC– poisoning, glucuronidation of AC might be possible in cats. It is however not the sole conjugation pathway for feline metabolism. Several new sulfate conjugates were detected in feline urine, as well as dechlorinated and oxidized AC metabolites.

## Supplementary Information

**Additional file 1.**

## Data Availability

The data underlying this article will be shared on reasonable request to the corresponding author.

## Abbreviations

AC: alpha-chloralose
°C: degrees Celsius
G: gram
HPLC: high performance/pressure liquid chromatography
Kg: kilogram
LC-MS/MS: liquid chromatography-tandem mass spectrometry
LC-HRMS/MS: liquid chromatography-high resolution tandem mass spectrometry
M: molar
µg: microgram
mg: milligram
µL: microlitre
mL: millilitre
mM: millimolar
MS: mass spectrometry
m/z: mass-to-charge-ratio
N_2_ flow: nitrogen gas flow
ng: nanogram
NVI: Norwegian Veterinary Institute
pH: potential of hydrogen
ppm: part per million (milligrams per litre)
Rcf: relative centrifugal force
Rpm: revolutions per minute
SVA: Swedish National Veterinary Institute (Statens Veterinärmedicinska Anstalt)
SLU: Swedish University of Agricultural Sciences
UDS: University Animal Hospital, Swedish University of Agricultural Sciences
UHPLC-MS/MS: ultra-high performance liquid chromatography tandem mass spectrometry
v/v: volume per volume
W: Watt
