# Supplementary material for: Alpha-chloralose poisoning in cats in three Nordic countries - the importance of secondary poisoning
Source: BMC Vet Res. 2022 Sep 5;18:334. doi: 10.1186/s12917-022-03370-w (PMC9446805; doi:10.1186/s12917-022-03370-w)
Supplement: Supplementary file 1 — Additional file 1. [file 12917_2022_3370_MOESM1_ESM.pdf]

Supplemental Material, Table of contents

**SI1**	Sample preparation of ingested mice
**SI2**	UHPLC-MS/MS instrument conditions
**SI3**	UHPLC-HRMS instrument conditions
**Fig. S1**	Calibration curve for standard addition method applied for sample A
**Fig. S2**	Calibration curve for standard addition method applied for sample B
**Fig. S3**	The UHPLC–HRMS/MS product ion spectrum from HCD of the $[M\text{-}H]^-$ ion of α-chloralose
**Fig. S4**	The UHPLLC–HRMS/MS product ion spectrum from HCD of the $[M\text{-}H]^-$ ion of the main isomer of tentatively identified chloralose glucuronide
**Fig. S5**	The UHPLC–HRMS/MS product ion spectrum from HCD of the $[M\text{-}H]^-$ ion of the tentatively identified 1,2-O-[(1R)-2,2-Dichloroethane-1,1-diyl]-α-D-glucofuranose
**Fig. S6**	The UHPLC–HRMS/MS product ion spectrum from HCD of the $[M\text{-}H]^-$ ion of the main isomer of tentatively identified chloralose sulfate
**Fig. S7**	The UHPLC–HRMS/MS product ion spectrum from HCD of the $[M\text{-}H]^-$ ion of the main isomer of tentatively identified oxidized chloralose

SI1. UHPLC-MS/MS instrument conditions

Urine samples were analysed on Agilent 6470A triple stage quadrupole (TSQ) instrument interfaced with Agilent 1290 Infinity II UHPLC System (Agilent Technologies, Santa Clara, CA, USA). One microliter of each sample was injected into Agilent Eclipse Plus C18 RRHD LC column (1.8 µm, 3x50 mm, Agilent Technologies, Santa Clara, CA, USA). Mobile phase solvent A was 10 mM ammonium acetate in water and B was MS grade acetonitrile. The column was kept at 30°C and the following gradient elution was used: start with 10% B and hold for 2 min, rising to 50% over 5 min, keep at 97% for 2 min followed by equilibration at 10% B for another 2 min. Flowrate was set at 0.6 mL/min across the LC run. Two multiple reaction monitoring transitions were monitored per analyte: chloralose 306.96→161 (collision energy (CE) was 15) for quantification and 306.96→101 (CE was 15) for confirmation. Coumachlor was used as internal standard and 341.1→284 (CE was 23) and 341.1→161 (CE was 23) transitions were used for quantification and confirmation respectively. Other instrument parameters were gas temperature: 250°C, gas flow: 5 L/min, nebulizer: 350 psi, sheath gas temperature: 350°C, sheath gas flow: 10 L/min, capillary voltage: 4.0 kV, nozzle voltage 500 V. Peak areas of internal standard coumachlor and both anomers of chloralose were collected in Agilent MassHunter Qualitative Analysis Navigator version. B.08.00.

For semi-quantitative analysis of chloralose in urine, peak areas of the α-anomer was plotted against calibration curve constructed for set of standard solutions of chloralose (α-chloralose purity of ≥80%, Sigma–Aldrich, St. Louis, MO, USA) in water covering a concentration range from 0.1 µg/mL to 100 µg/mL.

SI2. UHPLC-HRMS(/MS) instrument conditions

The ultra-high performance liquid chromatography high resolution mass spectrometry (UHPLC-HRMS) analysis was performed on a Vanquish Horizon UHPLC instrument (Thermo Fisher Scientific, Waltham, MA, USA) connected to a Q-Exactive mass spectrometer (Thermo Fisher Scientific, Waltham, MA, USA), equipped with a HESI-II heated electrospray interface. Aliquots (3 µL) of the samples were separated on an Agilent Eclipse Plus C18 RRHD LC column (1.8 µm, 3x50 mm, Agilent Technologies, Santa Clara, CA, USA). Liquid chromatography mobile phase solvent A consisted of 10 mM ammonium acetate in water and solvent B was MS grade acetonitrile. Analytes were eluted with a gradient utilizing mobile phases A and B. Solvent B ratio was set to 5% for the first minute and increased to 50% B over 2.5 min, followed by a column flush with 95% B for 1 min and then returned to 5% B and equilibration for 1 min. The flow rate was 0.5 mL/min for the whole gradient (6 min). For full-scan data acquisition, the mass spectrometer was set to scan the mass range of 200–1,000 m/z with the mass resolution set to 140,000 (at 200 m/z) in negative ionization mode. All HRMS/MS analyses were performed in the parallel reaction monitoring (PRM) mode using an isolation width of 0.4 m/z and applying higher-energy collision dissociation (HCD) of 25 eV, with resolution set to 70,000 (at 200 m/z). Other instrumental parameters were identical for FullMS and PRM and included a target ion count automatic gain control of 1×10^6, a maximum ion inject time of 256 ms, a S-lens voltage of 50 V, and an ESI voltage of −3.5 kV. Resulting FullMS .raw files were loaded to Compound Discoverer software v. 3.1 (Thermo Fisher Scientific, Waltham, MA, USA).

SI3. Sample preparation of ingested mice

Frozen samples were cut with a knife to approximately 1 g pieces and roughly homogenised by a household stainless steel coffee grinder (300 W). Two subsamples (A, 2.02 g and B, 2.21 g) were suspended in 5 mL of acetate buffer (0.1 M, pH 5.0) and internal standard was added (coumachlor, 100 µL of a 10 µg/mL methanol solution). Resulting mixtures were homogenized further using an Ultra-Turrax T25 mixer (IKA®-Werke GmbH & Co. KG, Staufen, Germany) at 15,000 rpm for one min. Five aliquots (1-5, 0.6 mL) of each subsample (A and B) were transferred to 1.5 mL polypropylene tubes and corresponding volumes of methanol solution of chloralose were added (0, 5, 12.5, 25 and 35 µL of 2500 µg/mL for sample A1-A5 and 0, 20, 40, 70 and 100 µl of 100 µg/mL for sample B1-B5). Samples A1-A5 and B1-B5 were vortexed for one minute and centrifuged at 3,000 rcf for five minutes. Clean supernatants were transferred into new tubes containing 0.6 mL of chloroform, vortexed for one minute and phase separated by centrifuging at 3000 rpm for 5 minutes. Bottom layers were transferred to new tubes, dried on N_2 flow at 60°C and resuspended in acetonitrile and 10 mM ammonium acetate in water (1:3, v/v). Suspensions were filtered throw Spin-X filters (10,000 rcf, 2 min) and transferred to HPLC vials. Samples were analysed on LC-MS/MS instrument (detailed conditions are described in SI1) and amount of chloralose in sample A and B was found by constructing corresponding calibration curves (Fig S1 and S2).

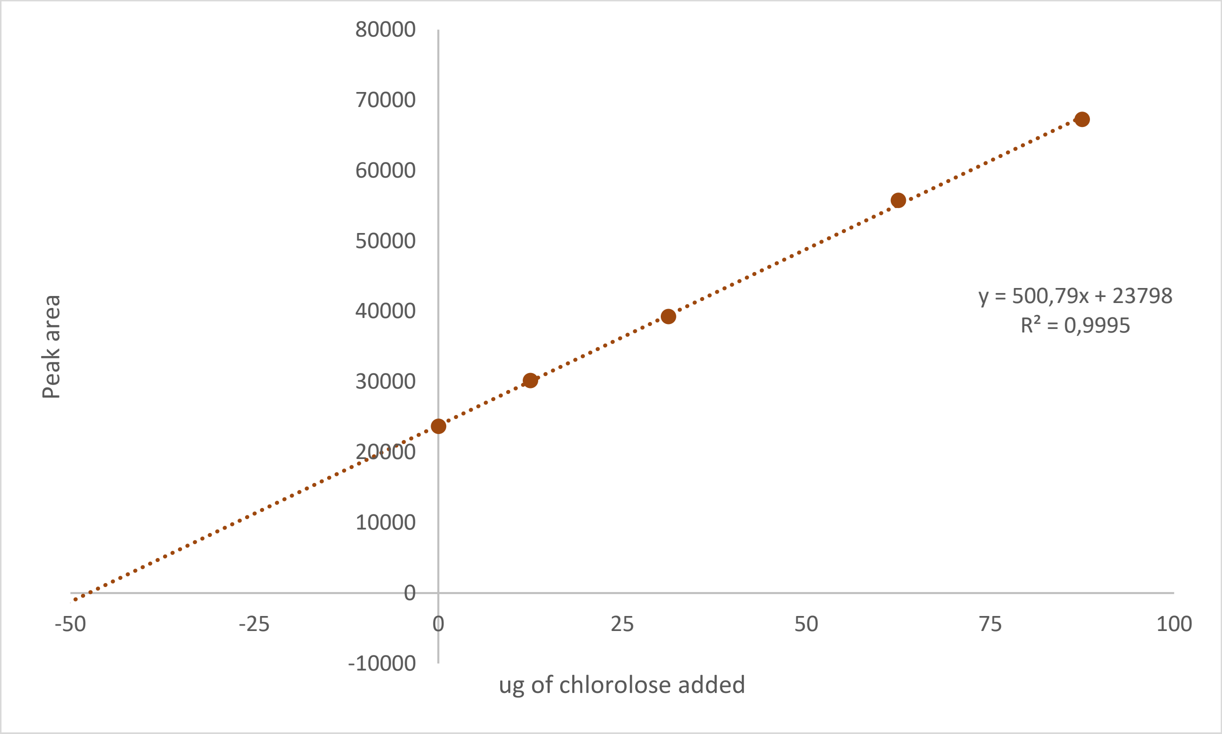

Fig S1. Calibration curve for the standard addition method applied on sample A.

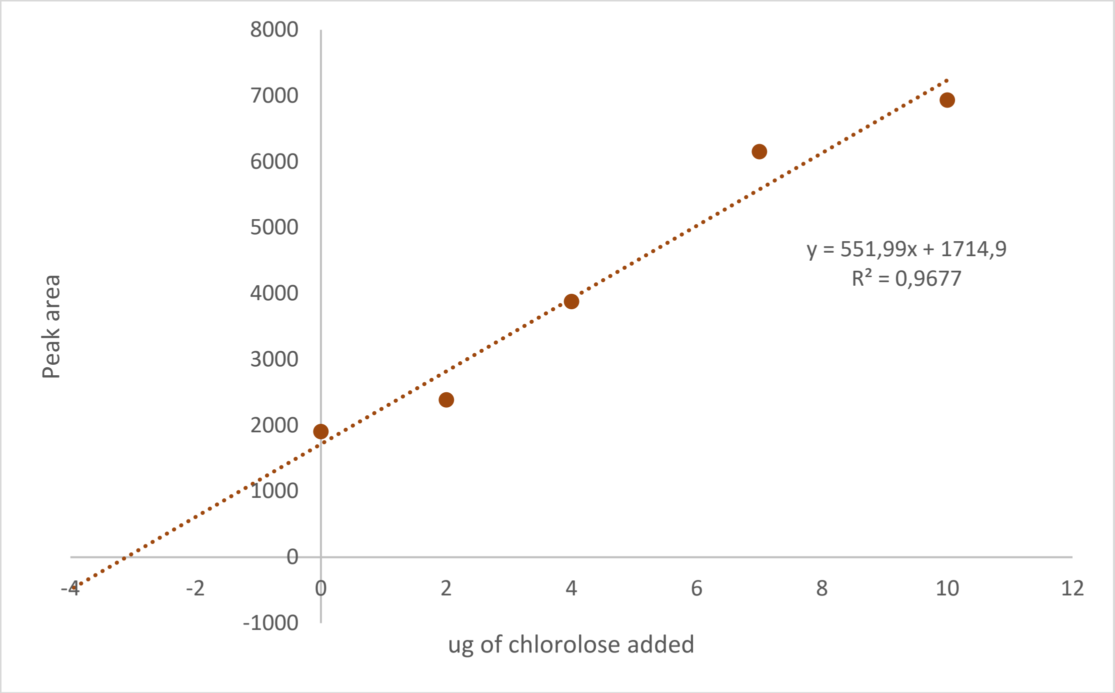

Fig S2. Calibration curve for the standard addition method applied on sample B.

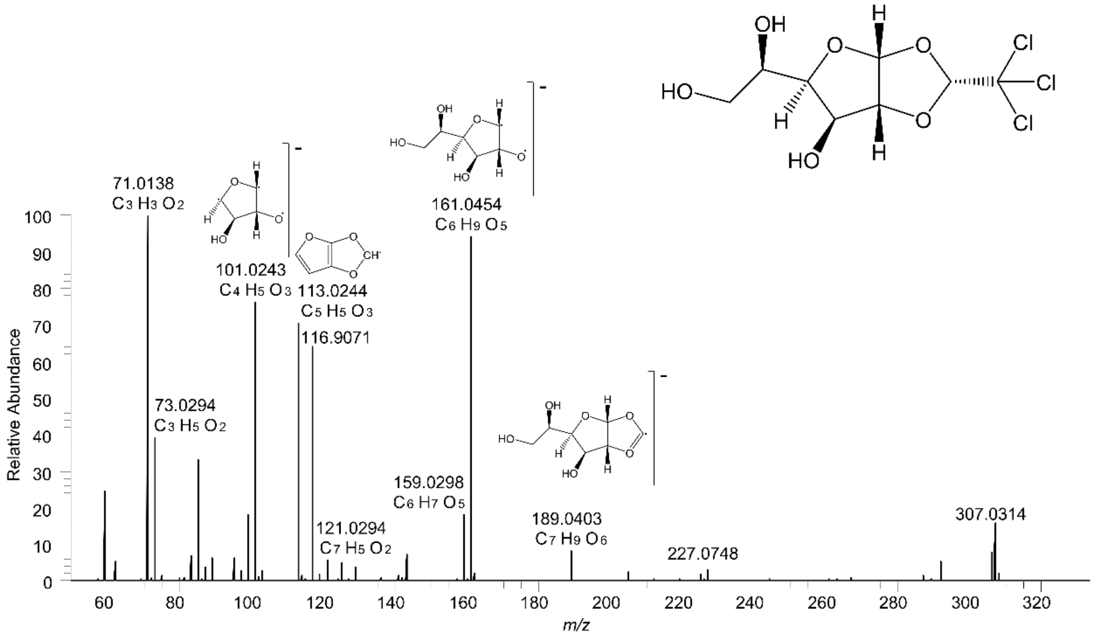

Fig S3. The UHPLC–HRMS/MS product ion spectrum from HCD of the [M-H]$^-$ ion of α-chloralose (RT=3.01 min, *m/z* 306.9548) showing main fragmentation pathways.

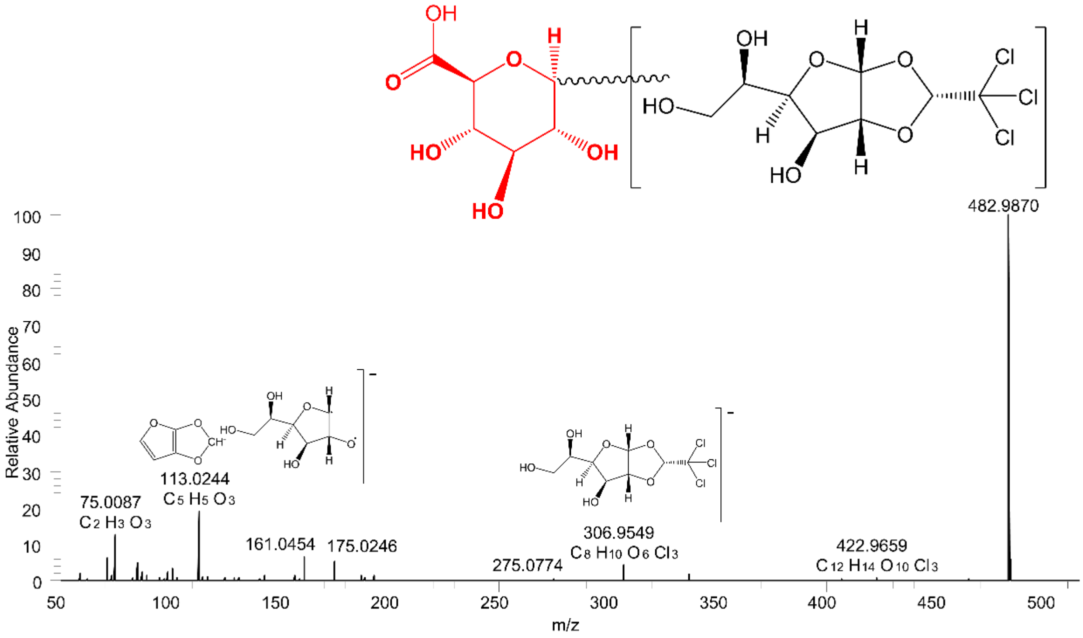

Fig S4. The UHPLC–HRMS/MS product ion spectrum from HCD of the [M-H]$^-$ ion of the main isomer of tentatively identified chloralose glucuronide (RT=2.84 min, *m/z* 482.9869). Note that position of glucuronic acid (shown in red) could not be assigned based on this spectrum.

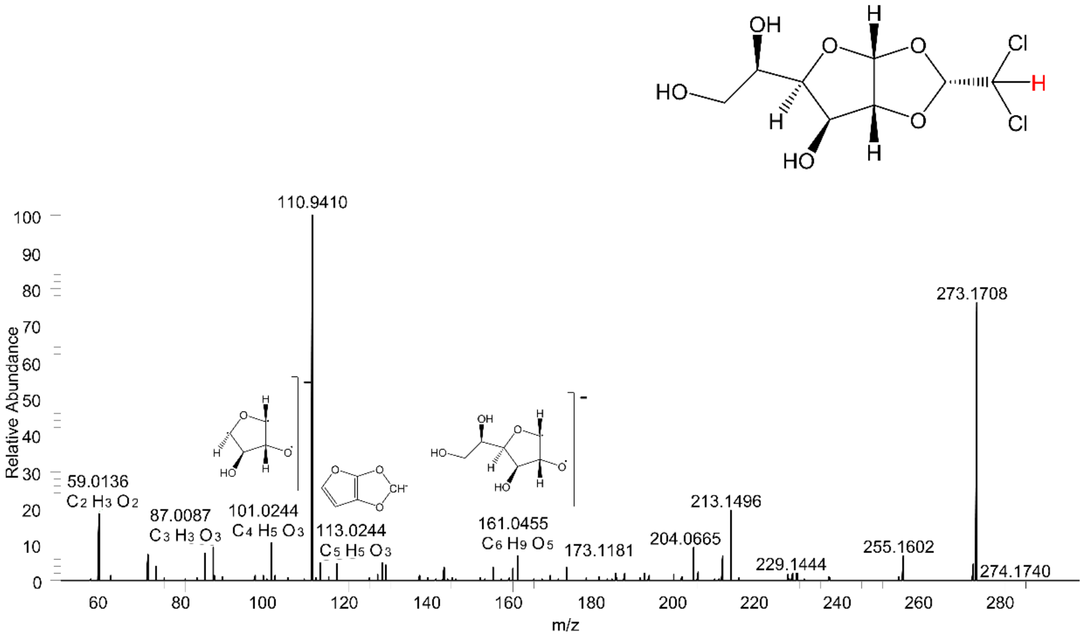

Fig S5. The UHPLC–HRMS/MS product ion spectrum from HCD of the $[M-H]^-$ ion of the tentatively identified 1,2-O-[(1R)-2,2-Dichloroethane-1,1-diyl]-α-D-glucofuranose (RT=3.04 min, *m/z* 320.9341). The position of the modification (shown in red) can be assigned based on this spectrum.

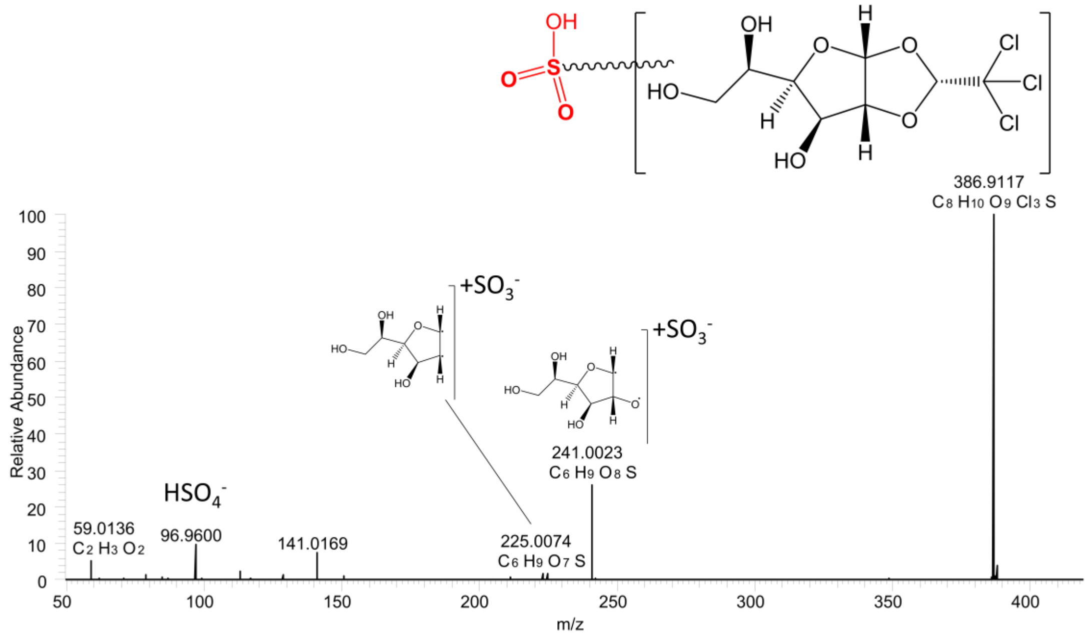

Fig S6. The LC–HRMS/MS product ion spectrum from HCD of the [M-H]$^-$ ion of the main isomer of tentatively identified chloralose sulfate (RT=2.77 min, *m/z* 386.9117) . Note that the position of the sulfate moiety (shown in red) could not be assigned based on this spectrum.

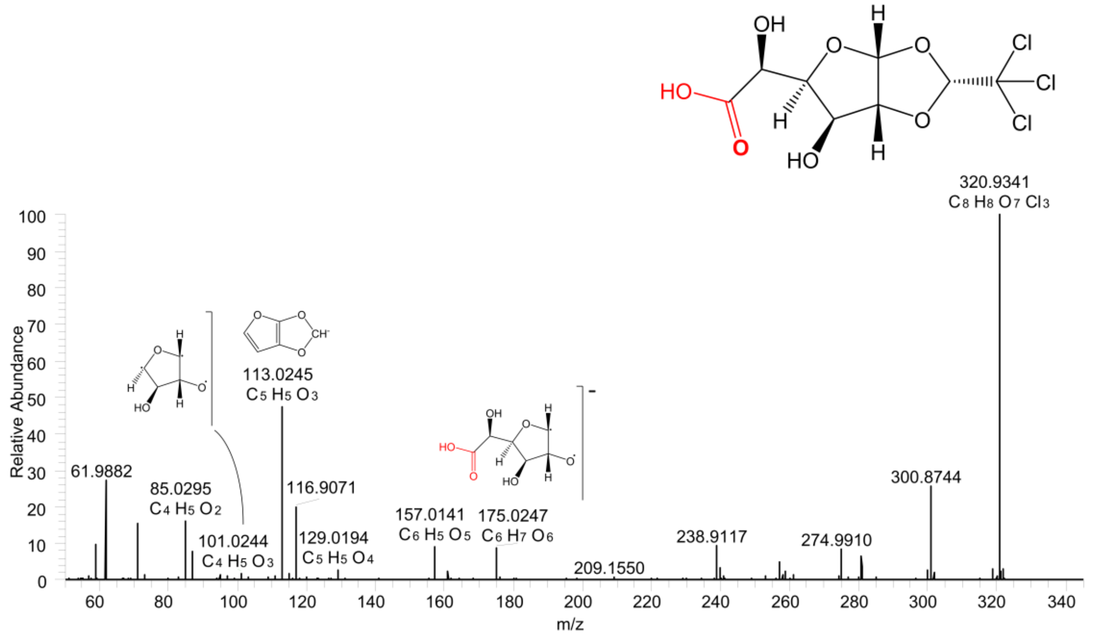

Fig S7. The UHPLC–HRMS/MS product ion spectrum from HCD of the [M-H]$^{-}$ ion of the main isomer of tentatively identified oxidized chloralose (RT=2.03 min, *m/z* 272.9938) showing main fragments. The position of the oxidation (shown in red) can be assigned based on this spectrum.
